# Supplementary material for: An Integrative Multi-Omics Workflow to Address Multifactorial Toxicology Experiments
Source: Metabolites. 2019 Apr 24;9(4):79. doi: 10.3390/metabo9040079 (PMC6523777; doi:10.3390/metabo9040079)
Supplement: Supplementary file 1 [file metabolites-09-00079-s001.zip › Supplymentary Materials_TableS1.pdf]

Supplementary Material

An integrative multi-omics workflow to address multifactorial toxicology experiments

Víctor González-Ruiz[1,2], Domitille Schvartz[2,3], Jenny Sandström[2,4], Julian Pezzatti[1,2], Fabienne Jeanneret[1,2], David Tonoli[1,2,§], Julien Boccard[1,2], Florianne Monnet-Tschudi[2,4], Jean-Charles Sanchez[2,3], Serge Rudaz[1,2 *]

[1] *Analytical Sciences, School of Pharmaceutical Sciences, University of Geneva, University of Lausanne, Geneva, Switzerland*
[2] *Swiss Centre for Applied Human Toxicology, Switzerland*
[3] *Translational Biomarker Group, Department of Internal Medicine Specialties, University of Geneva, Geneva, Switzerland*
[4] *Department of Physiology, University of Lausanne, Lausanne, Switzerland*
[§]*Present address: Clinical Research Centre, Geneva University Hospitals, Geneva, Switzerland*

** Corresponding author: Prof. Serge Rudaz. School of Pharmaceutical Sciences, University of Geneva, University of Lausanne, University Medical Centre, 1 Rue Michel-Servet, 1205 Geneva, Switzerland. E-mail: serge.rudaz@unige.ch*

Table S1: *ANOVA saliences of the AMOPLS model. The table shows which percentage of the variability of each OPLS predictive component can be attributed to each effect. Values marked in bold highlight effect having the largest contribution to each predictive components.*

	tp1	tp2	tp3	tp4	tp5	tp6	tp7	tp8	tp9	to
Maturation state	**98.6%**	0.7%	0.7%	0.7%	2.7%	3.9%	6.6%	5.3%	6.9%	7.8%
Exposure duration	0.2%	**91.8%**	1.0%	1.0%	3.7%	5.4%	9.1%	7.3%	9.5%	10.7%
TMT concentration	0.2%	1.3%	1.3%	**91.9%**	4.7%	6.9%	11.7%	9.4%	**23.1%**	13.7%
Maturation × Exposure duration	0.2%	1.2%	**91.7%**	1.2%	4.4%	6.4%	10.8%	8.7%	11.3%	12.7%
Maturation × Concentration	0.3%	1.7%	1.8%	1.8%	6.5%	**59.0%**	15.9%	**44.6%**	16.6%	18.7%
Exposure duration × Concentration	0.3%	1.6%	1.7%	1.7%	**71.5%**	8.8%	**29.9%**	12.0%	15.7%	17.6%
Residuals	0.3%	1.7%	1.8%	1.8%	6.5%	9.5%	16.1%	12.9%	16.8%	**18.9%**
