# Supplementary figures and images for: An Integrative Multi-Omics Workflow to Address Multifactorial Toxicology Experiments
Source: Metabolites. 2019 Apr 24;9(4):79. doi: 10.3390/metabo9040079 (PMC6523777; doi:10.3390/metabo9040079)

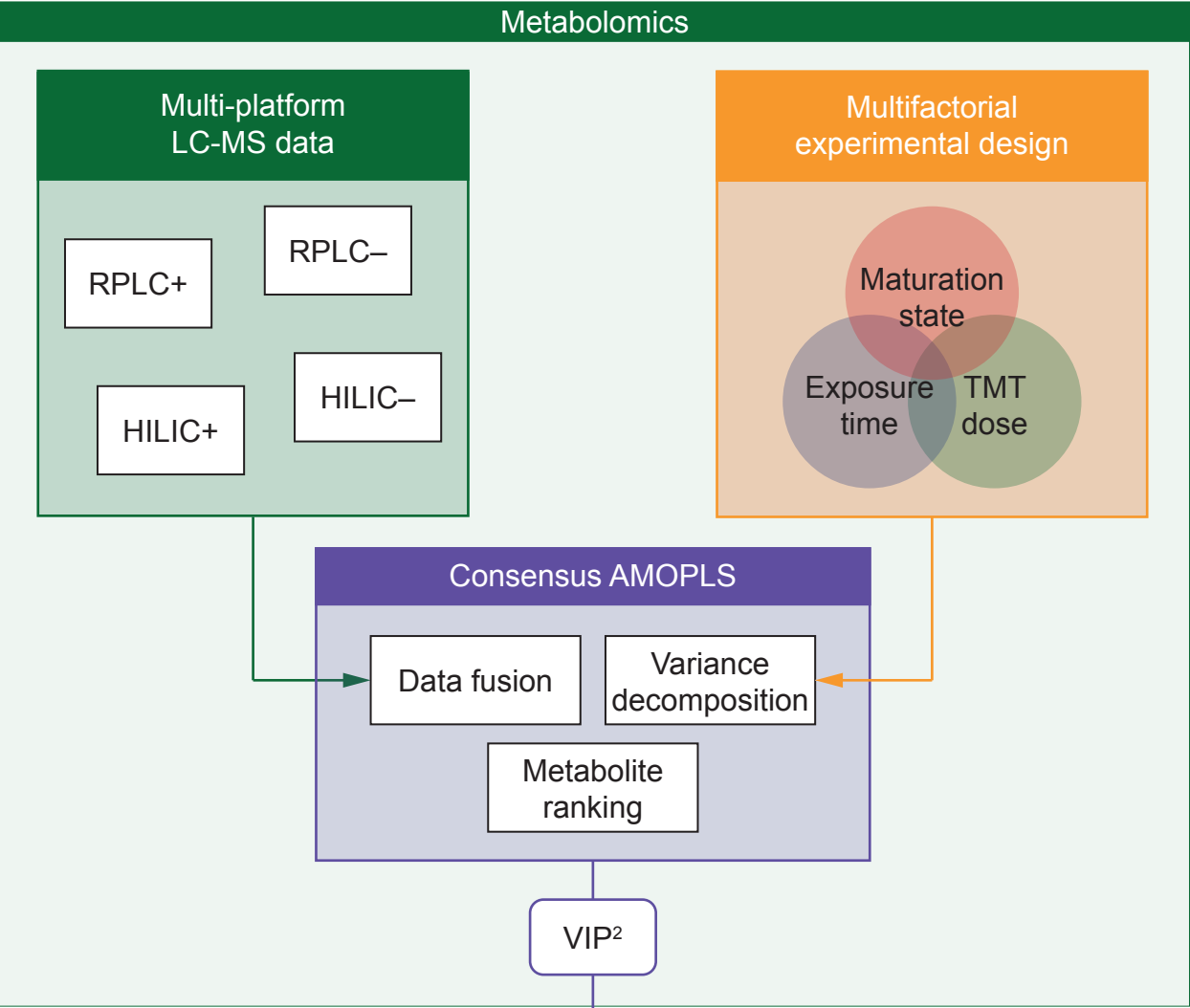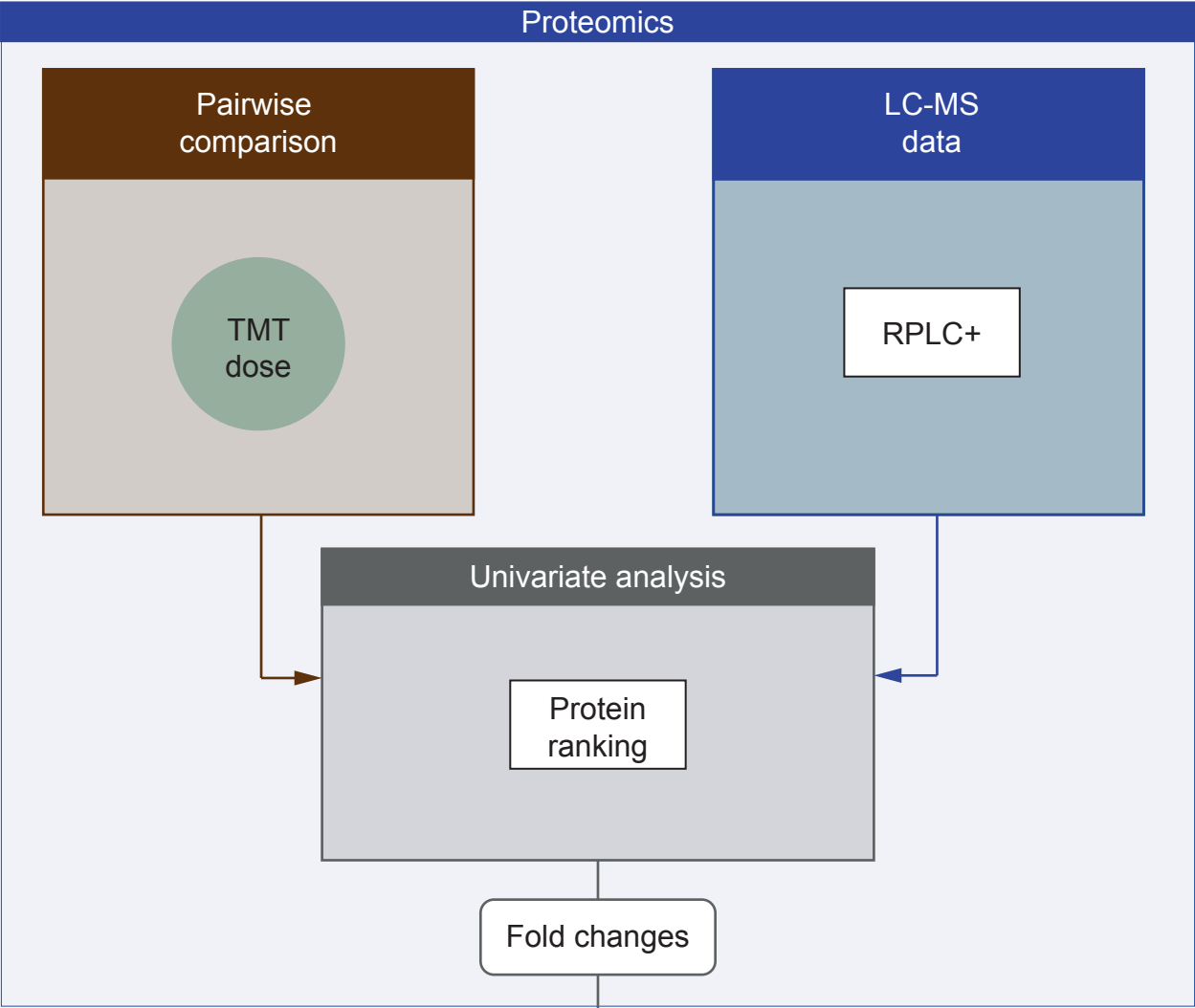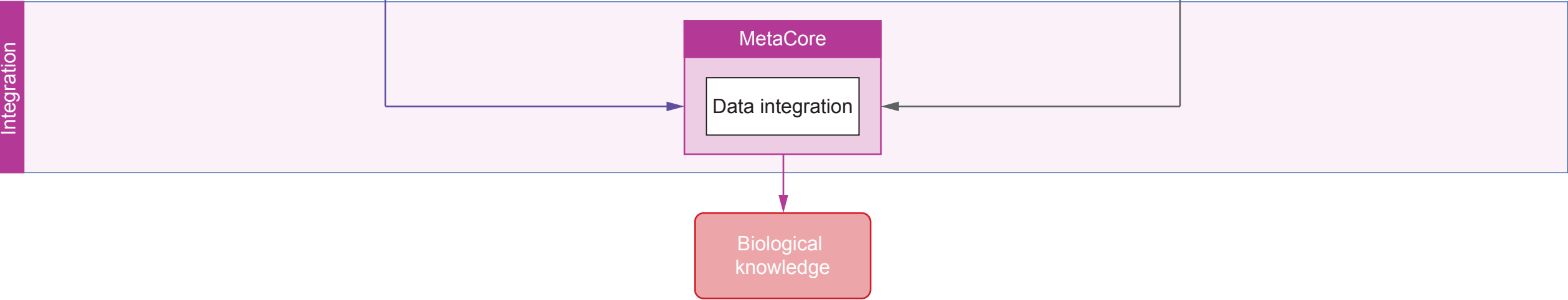

Supplement: Supplementary file 1 [file metabolites-09-00079-s001.zip › Supplymentary Materials_FigureS1.pdf]
